# Quantifying Fluorescently Labeled Ceramide Levels in Human Sarcoma Cell Lines in Response to a Sphingomyelin Synthase Inhibitor

**DOI:** 10.3390/mps2030076

**Published:** 2019-08-31

**Authors:** Srinath Pashikanti, Farjana Afrin, Trevor C. Meldrum, John L. Stegelmeier, Adriene Pavek, Yashar A. Habashi, Kaniz Fatema, Jared J. Barrott

**Affiliations:** Department of Biomedical and Pharmaceutical Sciences, Idaho State University, Pocatello, ID 83209, USA

**Keywords:** ceramide, synovial sarcoma, osteosarcoma, sphingomyelin synthase inhibitor, jaspine B, cellular assay

## Abstract

Sphingolipid metabolism is an important process in sustaining the growth needs of rapidly dividing cancer cells. Enzymes that synthesize sphingolipids have become attractive targets in cancer pharmacology. Ceramide is a precursor for synthesizing sphingolipids such as sphingomyelin, sphingosine-1-phosphate, and glucosylceramide. Sphingomyelin synthase (SMS) is the enzyme that transfers a phosphatidylcholine to ceramide to generate sphingomyelin. To test the inhibition of SMS, scientists assess the buildup of ceramide in the cell, which is cytotoxic. Because ceramide is a small lipid molecule, there are limited tools like antibodies to detect its presence. Alternatively, designated machines for small-molecule separation coupled with mass spectrometry detection can be used; however, these can be cost-prohibitive. We used a commercially available NBD-ceramide to apply to human cancer cell lines in the presence or absence of a known SMS inhibitor, jaspine B. After short incubation times, we were able to collect cell lysates and using solvent extraction methods, run the cellular material on a thin-layer chromatography plate to determine the levels of intact fluorescently labeled ceramide. Brighter fluorescence on the TLC plate correlated to greater SMS inhibition. Small molecules can then be screened quantifiably to determine the biological impact of inhibiting the sphingolipid metabolism pathways involving ceramide.

## 1. Introduction

**Figure 1:**
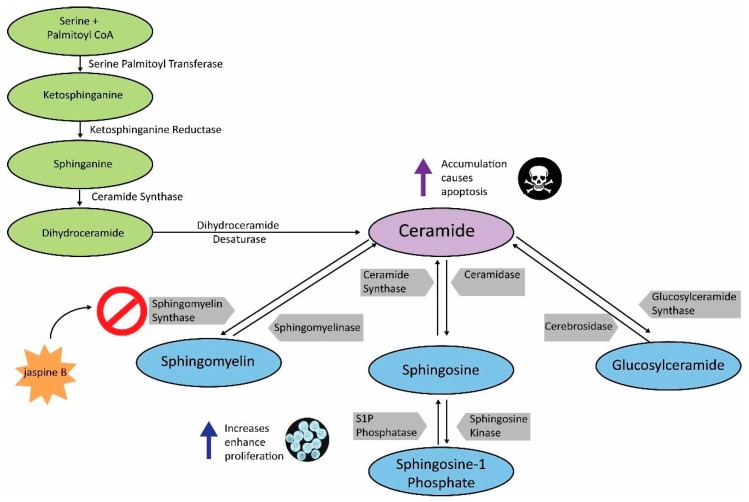
Sphingolipid metabolism pathway. Schematic demonstrating the key enzymes and substrates in sphingolipid metabolism. Increased levels of sphingosine-1 phosphate, glucosylceramide, and sphingomyelin enhance cell proliferation, while administration of jaspine B, a sphingomyelin synthase (SMS) inhibitor, results in ceramide accumulation and apoptosis. Inhibiting the sphingomyelin arm of ceramide metabolism can also result in ceramide being shunted to other sphingolipids such as sphingosine-1 phosphate and glucosylceramide.

Cancer is a disparate disease driven by numerous and diverse metabolic needs [1]. Sphingolipid metabolism is a pathway that is commonly upregulated in cancer and thus, has become a viable target for drug screening purposes [2,3]. Ceramide is central to sphingolipid metabolism and serves as a precursor to several functionally important sphingolipids [2]. Sphingomyelin synthase (SMS) 1 and 2 are essential enzymes in the conversion of ceramide to sphingomyelin [4]. Inhibition of sphingomyelin synthases results in an accumulation of ceramide, resulting in cytotoxicity and eventually, apoptosis (Figure 1) [5]. To prevent ceramide accumulation, cancer cells can divert ceramide to the synthesis of other sphingolipids. Sphingosine-1 phosphate and glucosylceramide have likewise demonstrated the ability to enhance tumorigenesis in several cancers, and are thus targets for cancer therapies [6,7]. Future combinations of these targeted therapies should result in increased apoptosis in cancer cells, both in vitro and in vivo. 

There are copious ways of measuring cell viability and events associated with apoptosis during pharmacological screening of small-molecule inhibitors [8,9], but these methods do not pinpoint a mechanism of action of how a cell arrived at that end fate. Understanding the molecular underpinnings of how a drug works will lead to more precise applications in precision medicine and circumvent future developments of resistance. To this end, researchers have sought to understand the biological relevance of SMS1 and SMS2 inhibitors in the context of the sphingolipid metabolism pathway [4,5].

Measuring ceramide levels is one of the most relevant outputs upon inhibition of SMS1 and SMS2 [10]. One approach is to use an antibody, for which there are only four commercial options derived from two hybridoma clones (Table 1). All four products are mouse monoclonal antibodies, thus limiting their applications in preclinical mouse models. Additionally, immunohistochemical and ELISA-based assays require multiple steps and often 1–2 days to complete the assays. Furthermore, antibody detection methods can be fraught with non-specific recognition, thus obscuring positive results. 

Other analytical techniques that are more accurate in detecting ceramide levels include liquid chromatography–tandem mass spectrometry (LC/MS) such as HPLC, LC-ESI-MS/MS, HPLC-MSMS-MRM [11,12,13]. These methods are more commonly used to measure small molecules, such as sphingolipids and their relative quantities. However, the sample preparation can be laborious, the liquid chromatography separation can be time-consuming, the chemical preparation of samples can result in sample loss thus creating run-to-run variation, and equipment costs and maintenance could be prohibitive [13]. 

## 2. Experimental Design

We have developed a rapid screening method to detect levels of ceramide in the presence of an SMS inhibitor. This approach relies on the commercially available C6-NBD ceramide (N381205, North York, ON, Canada). This is a fluorescently labeled ceramide analogue that is permeable to cell membranes. Once internalized, the fluorescently labeled ceramide incorporates readily into membranes that comprise the Golgi apparatus, the endoplasmic reticulum, and the nuclear envelope [10]. 

**Figure 2:**
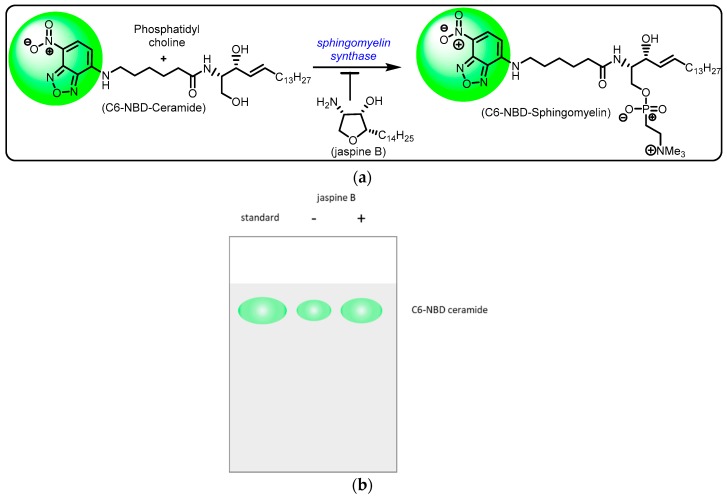
Schematic representation of sphingomyelin synthase inhibition by jaspine B. (**a**) Sphingomyelin synthase catalysis involves conversion of ceramide to sphingomyelin utilizing the cofactor phosphatidylcholine. The marine natural product, jaspine B, exhibits sphingomyelin synthase inhibition, thus resulting in unchanged C-6 NBD ceramide fluorescence. (**b**) Hypothetical results after thin-layer chromatography to show that the administration of jaspine B reduces the fluorescent presence of C6-NBD ceramide.

In our assay, we applied the C6-NBD ceramide to live cells with or without the SMS inhibitor, jaspine B (Figure 2) [14]. Following shorter incubation times and higher concentrations of C6-NBD ceramide compared to previously reported experiments [10], cell lysates were extracted. The extract was mixed briefly with a precipitating solvent and blotted immediately to a silica gel for thin-layer chromatography (TLC). Apart from differing in the cell types, short incubation times, and higher ceramide concentrations, the extraction method did not focus on solubilization of the lipid fraction like similar protocols [10]. Cell lysates were eluted and underwent separation of cellular molecules based on the polarity. Cells incubated with C6-NBD ceramide were eluted along the TLC plate and imaged in an Azure c600 imaging station for fluorescence using GFP excitation and emission filters (488/520 nm). Levels of fluorescence were easily quantified and an inverse relationship between the level of fluorescence detected and the inhibition of SMS was determined. This method allows for simple and rapid identification of potent inhibitors of SMS by measuring the presence of exogenously added fluorescently labeled ceramide to live human cancer cells.

### 2.1. Materials

- C-6 NBD Ceramide (Toronto Research Chemicals, North York, ON, Canada; Cat. no.: N381205)
- Jaspine B (Pashikanti lab, Pocatello, ID, USA)
- SJSA-1 (ATCC, Manassas, VA, USA; Cat. no.: CRL-2098)
- U2-OS (ATCC, Manassas, VA, USA; Cat. no.: HTB-96)
- RPMI 1640 Medium (Caisson Labs, Smithfield, UT, USA; Cat. no.: RPL04-6)
- Fetal Bovine Serum (Atlanta Biologicals, Flowery Branch, GA, USA; Cat. no.: S11150)
- Penicillin/Streptomycin (Atlanta Biologicals, Flowery Branch, GA, USA; Cat. no.: B21110)
- Trypsin 0.25% EDTA (Atlanta Biologicals, Flowery Branch, GA, USA; Cat. no.: B81310)
- NaCl (Sigma Chemical Company, St. Louis, MO, USA; Cat. no.: S-9625)
- NP-40 (Fluka Analytical, Mexico City, Mexico; Cat. no.: 74385)
- Phosphate Buffered Saline (Genesee Scientific, El Cajon, CA, USA; Cat. no.: 25-508)
- Pyridine (Alfa Aesar, Haverhill, MA, USA; Cat. no.: A12005-AP)
- Silica gel plates TLC-G (Silicycle, Quebec, QC, Canada; Cat. no.: TLG-R10014BK-323)
- Toluene (ThermoFisher Scientific, Waltham, MA, USA; Cat. no.: S25611A)
- Tris HCl (ThermoFisher Scientific, Waltham, MA, USA; Cat. no.: EC 201-064-4)

### 2.2. Equipment

- Azure c600 Imaging Station (Azure Biosystems, Dublin, CA, USA; Cat. no.: c600)
- Countess II (ThermoFisher Scientific, Waltham, MA, USA; Cat. no.: AMQAX1000)
- Countess Slides (ThermoFisher Scientific, Waltham, MA, USA; Cat. no.: C10283)
- MiniSpin (Eppendorf, Hamburg, Germany; Cat. no.: 22620100)
- Spectrafuge 6C (Labnet, Edison, NJ, USA; Cat. no.: LI-CF-SF6C)
- EVOS FL (ThermoFisher Scientific, Waltham, MA, USA; Cat. no.: AMF4300)
- FORMA SERIES II CO₂ Incubator (ThermoFisher Scientific, Waltham, MA, USA; Cat. no.: 31300)

## 3. Procedure

### 3.1. Preparing Single Cell Suspensions

- Wash adherent cancer cells growing in a tissue-culture-treated flask with a surface area of 75 cm^2^ with 7 mL of 1 X phosphate buffered saline (without Ca^2+^ and Mg^2+^). Remove phosphate buffer saline.
- Add 2 mL of pre-warmed 0.25% Trypsin-EDTA to each flask and return to the tissue culture incubator with the following conditions of 37 °C and 5% CO_2_ and incubate for 5 min or until most cells have detached from the bottom of the flask.
- Quench the trypsin by adding 2 X the volume (4 mL) of cell culture medium. Pipette the 6 mL solution up and down, pointing the tip towards the side of the flask on which the cells were growing.
- **OPTIONAL STEP.** DMEM or RPMI supplemented with 10% fetal bovine serum and penicillin/streptomycin are commonly used cell culture mediums for these cells, but this can be adaptable as long as a solution with proteins is used to quench the trypsin.
- Transfer the 6 mL of single cell suspensions to a 15 mL conical tube and centrifuge the solution at 1800× *g* and room temperature for 4 min.
- Decant the media solution and resuspend the cell pellet in 4–5 mL of fresh cell culture medium.
- Count cell concentrations. Set aside 1−1.5 million cells for each treatment condition in its own 1.7 mL microcentrifuge tube.
- Centrifuge cells again at 1800× *g* for four minutes and resuspend cells in 20 μL of phosphate-buffered saline.

### 3.2. Treating Cells with an SMS Inhibitor and Adding C6-NBD Ceramide

- For each cell line, test two conditions. Prepare a concentration of jaspine B, an SMS inhibitor, at 5 μM.
- **OPTIONAL STEP.** We prepare our stock solutions at a concentration of 10 mM in DMSO. Dilution to a working concentration of 5 μM is achieved with phosphate-buffered saline.
- To test the inhibition of SMS, add either 2 μL of 5 μM jaspine B or 2 μL of phosphate-buffered saline to the labeled microcentrifuge tubes containing 1−1.5 million cells in 20 μL of solution. This will dilute the jaspine B to a final concentration of 500 nM.
- Add 2 μL of 100 μM C6-NBD ceramide to all the cell solutions, both treated and control. Thus resulting in a final concentration of 10 μM.
- Incubate at 37 °C for 30 min.
- Centrifuge the cells and solution at 1800× *g* for four minutes and, using a micropipette, remove all liquid.
  **⦷ PAUSE STEP.** Cell pellets can be stored at −20 °C for later analysis.
- Add 50 μL of phosphate-buffered saline and pipet up and down to wash the cells.
- Centrifuge the cells and solution at 1800× *g* for four minutes and using a micropipette remove all liquid.

### 3.3. Lysing Cells and Sample Preparation for Running Thin-Layer Chromatography

- Incubate cells in 20 μL of mild cell lysis buffer for 10 min.
  NOTE: Mild cell lysis buffer consisted of 10 mM Tris-HCl pH 8.1, 10 mM NaCl, 0.5% NP-40.
- Clarify cell lysates by centrifuging at >10,000× *g* for 10 min.
- Collect the supernatant fraction and add 20 μL of 100% methanol.

### 3.4. Running the Thin-Layer Chromatography

- Load 40 μL of each sample by applying the liquid to a single location near the bottom of a silica gel plate.
- Include a 2 μL standard of C6-NBD ceramide in 20 μL of methanol that will allow to detect where the unmodified C6-NBD ceramide is located on the gel.
- Place the plate in a beaker that has a solvent (Toluene: Pyridine:Water – 46:46:8) just below the blotted samples.
- Allow the solvent to carry the samples up the silica gel by capillary action.
- Remove the silica gel and allow to air dry.

### 3.5. Imaging Samples on a Fluorescent Imaging Station

- Using a fluorescent imaging station, expose the sample to 488 nm wavelength of light for excitation, then capture the emission with 520 nm wavelength filters.
- **OPTIONAL STEP.** The Azure c600 imaging system takes simultaneous images with blue, green and red filters. Fluorescence could be detected with both blue and green filters.

### 3.6. Analyzing the Images

**Figure 3:**
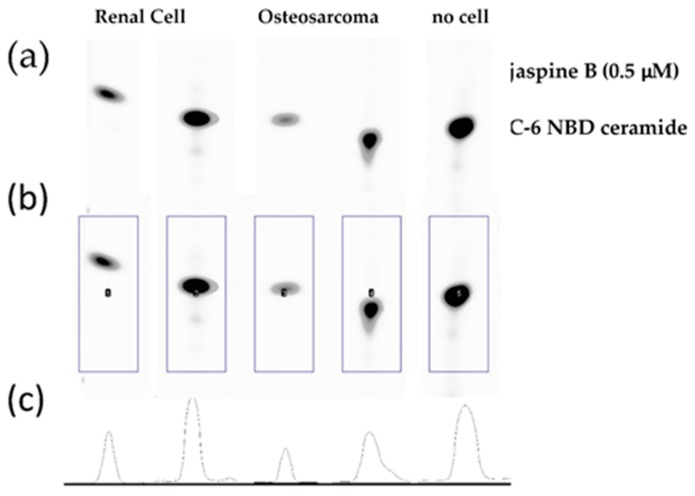
Steps in identifying regions of interest of C-6 NBD ceramide on a TLC plate. (**a**) Raw TIF file converted to gray scale with white background; (**b**) selection of regions of interest (ROIs) using the rectangle tool in FIJI; (**c**) generation of densitometry curves and measuring the area under the curve corresponding to the blot size and intensity.

**Figure 4:**
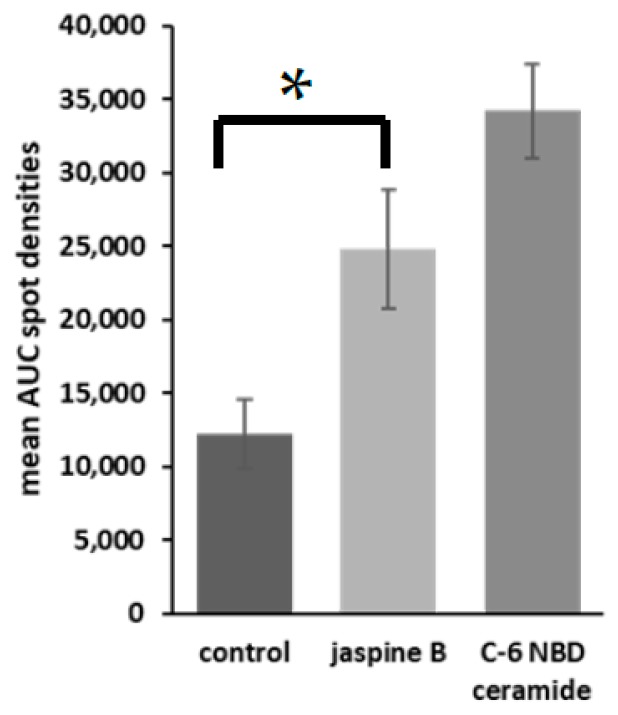
Representative bar graphs of fluorescent densitometry. U2-OS, SJSA-1, hFOB, and FUUR-1 were treated with either 0.5 μM jaspine B or control for 30 min and the spot intensities were averaged together with standard deviations of the mean. These values were compared to C-6 NBD ceramide as the standard control. The unit of measurement is the area under the curve. The *p* value = 0.0017, *.

- Export TIF image files and open in FIJI software [15].
- Transform images to grayscale and invert the colors so as to have a white background with gray blots (Figure 3a).
- Create a rectangle box around the first blot and press 1 to mark as a region of interest. Drag the box, which creates a second region of interest, to the next blot and press 2 to continue marking regions of interest. Repeat for all blots on the silica gel by pressing 2 until the last blot and then press 3. This generates area under the curve images (Figure 3b,c).
- Select the wand tool in FIJI and click in the middle of the area under the curve. This measures the value and puts it in a separate window.
- Transfer to a spreadsheet document and perform statistical analyses to compare the control versus the treated samples (Figure 4).

## 4. Expected Results

**Figure 5:**
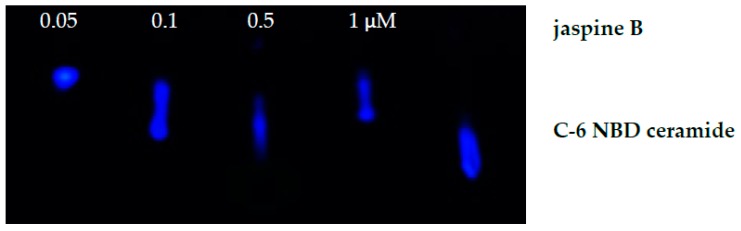
Fluorescent image of C-6 NBD ceramide on a thin-layer chromatography plate. Synovial sarcoma cells were treated with serial dilutions of jaspine B (0.05–1.0 μM) for 30 min and compared to the standard of C-6 NBD without cells and no jaspine B treatment.

The methods outlined provide a rapid detection of inhibition of enzymes linked to ceramide metabolism. Using the SMS inhibitor, jaspine B, we observed an increased presence of unmodified C-6 NBD ceramide when these cells were treated with 0.5 μM of jaspine B (Figure 3). Additionally, cells were treated with a range of serial dilutions of jaspine B and demonstrated effective inhibition of C-6 NBD ceramide across the concentration range of 0.1−1.0 μM (Figure 5). These observations were made after separating cellular lysates by thin-layer chromatography and imaging for fluorescence on a fluorescent imaging station. In cells that have increased levels of SMS, the C-6 NBD ceramide is metabolized presumably into sphingomyelin. However, inhibiting SMS allows for ceramide to persist in the cell unchanged. The fluorescent spots are easily quantifiable using software that can convert size and intensity into areas under the curve. 

**Figure 6:**
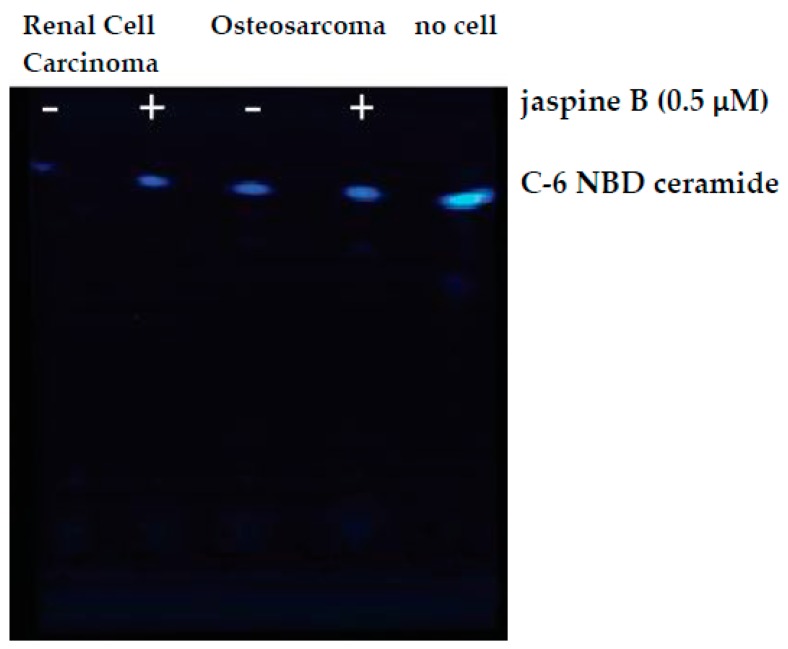
Raw image output from Azure c600 imaging station. Renal cell carcinoma and osteosarcoma cells were treated with 0.5 μM jaspine B for 90 min and compared to no cell control of C-6 NBD ceramide. TLC silica plate was imaged in an Azure c600 under blue, green, and red filters. The above image is an overlay of the three channels.

This method is an effective means of screening the biological consequence of inhibiting enzymes in the ceramide metabolism pathway. While this method has focused exclusively on the effects of inhibiting sphingomyelin synthase, there are multiple pathways involved in ceramide metabolism (Figure 1) and similar approaches have been used to investigate inhibitors of ceramidase and sphingosine kinase [2,3,6,7,8]. One limitation of the study is that because there are multiple pathways responsible for ceramide metabolism, inhibiting one arm of the pathway does not fully prevent the metabolism of ceramide, which could result in the loss of the fluorescent signal even in the presence of a potent and specific inhibitor. We found that shorter incubation times of 30 min with 100 μM C-6 NBD ceramide demonstrated the most dramatic effects between the treated and control samples (Figure 3). Conversely, when we allowed samples to incubate for 90 min, the differences appeared more subtle (Figure 6).

**Figure 7:**
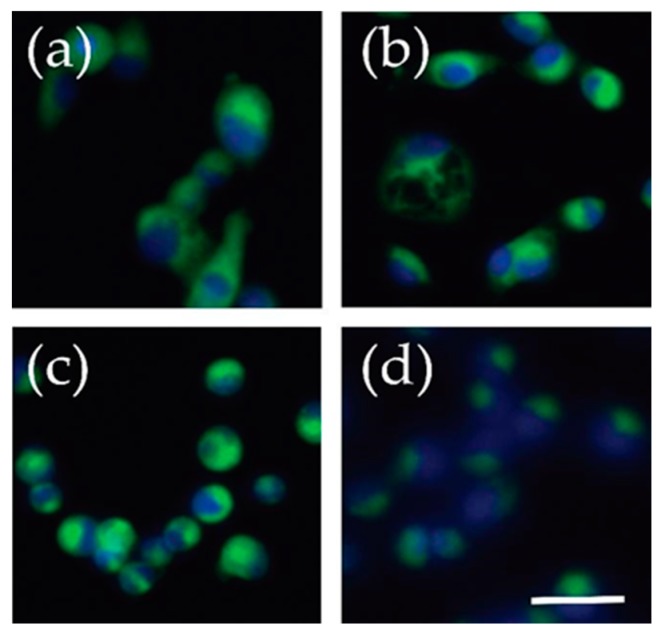
Fluorescent live cell imaging of C6-NBD ceramide. Cells were incubated with C-6 NBD ceramide for 30 min while still adherent and imaged with an EVOS FL microscope. The green represents the C-6 NBD ceramide and blue is a result of a NucBlue stain marking the nuclei. (**a**) U2-OS, osteosarcoma (**b**) SJSA-1, osteosarcoma, (**c**) Yamato, synovial sarcoma (**d**) FUUR-1, renal cell carcinoma. Scale bar = 50 μm.

While our work was performed on adherent cells that readily internalized the C-6 NBD ceramide (Figure 7), we preferred working with these cells in suspension after trypsinization because it minimized the volume of working solutions while maximizing the cells to perform the experiment. We considered working with cells adhered to a 96-well plate to minimize the volume and perform more high-throughput analysis. However, cell numbers were limited to 50,000 cells per well and the presence of a fluorescent signal does not indicate the metabolism of ceramide, as ceramide could be metabolized and the fluorescent metabolite could still persist in the cell. To circumvent this issue, we thought it was necessary to separate the cell lysate by TLC to see how much of the fully intact C-6 NBD ceramide was present. We expected to see multiple-size fluorescent bands, especially in our control cell lysates, but we were never able to find a metabolized product, just a diminished signal in the presence of an uninhibited SMS enzyme.

Lastly, this method is preferable from a safety standpoint. Ten to fifteen years ago, radiolabeling was prominent in sphingolipid molecular research [16,17]. However, working with radiolabeled materials poses minor health risks and increases federal and institutional scrutiny [18]. The use of a fluorescently labeled ceramide is a safer alternative, without comprising the integrity or sensitivity of the assay. As such, more researchers are changing to fluorescently labeled small molecules within basic sciences [19,20]. Not only is fluorescently labeled ceramide commercially available, but several of the sphingolipids are being produced with fluorescent tags to facilitate safer and sensitive detection of these lipid molecules to implement in biochemical experiments [21,22,23].

## 5. Reagents Setup

Jaspine B was prepared and stored at 4 °C as a 10 mM concentration in DMSO. Jaspine B was prepared at a 10 × concentration of 5 μM in PBS as a working concentration. C-6 NBD ceramide was shipped at room temperature and reconstituted at 100 μM in DMSO and then stored at −20 °C.

## Figures and Tables

**Table 1:** List of anti-ceramide monoclonal antibodies.

Company	Catalog	Hybridoma Clone #	Applications
United States Biological	C2777-95	6D311	AP, ELISA, FACS, IHC-F/P
Epigentek	A-0549-001	MID15B4	ELISA, ICC, IHC-F/P
Enzo Life Sciences	ALX-804-196-T050	MID15B4	ELISA, FACS, ICC, IHC-F/P
Lifespan Biosciences	LS-C79075	MID15B4	ELISA, FACS, ICC, IHC-F

AP—affinity purification; ELISA—enzyme-linked immunosorbent assay; FACS—fluorescence-activated cell sorting; IHC—immunohistochemistry; ICC—immunocytochemistry; F—frozen; P—paraffin.
